# An Electric Plant

**Published:** 1891-02

**Authors:** 


AN ELECTRIC PLANT.—A plant has lately been discovered in India which is possessed of a high degree of electrical power. By breaking a leaf off, the hand is said to receive a shock about equal to that produced by the conductor of an induction coil. A magnetic needle is affected by it at six inches. The electric power of the plant is stated to reach its maximum in the afternoon and to disappear at night. It is much increased during a storm. The soil on which the plant grows contains none of the magnetic metals, iron, cobalt or nickel.

---

